# Obesity and normal birth: A qualitative study of clinician’s management of obese pregnant women during labour

**DOI:** 10.1186/s12884-015-0673-2

**Published:** 2015-10-12

**Authors:** Angela Kerrigan, Carol Kingdon, Helen Cheyne

**Affiliations:** NMAHP Research Unit, University of Stirling, Stirling, FK9 4LA United Kingdom; School of Health, University of Central Lancashire, Preston, PR1 2HE United Kingdom

**Keywords:** Obesity, Normal birth, Intrapartum care, Challenges, Health professionals

## Abstract

**Background:**

Currently one-fifth of women in the UK are obese. Obese, pregnant woman are at an increased risk of experiencing complications of labour and serious morbidity. However, they are also more likely to undergo medical interventions such as induction of labour and caesarean section which in themselves confer additional health risks for obese women such as wound infection and deep vein thrombosis. Reducing unnecessary interventions and increasing normal birth rates for obese women would substantially improve their postnatal health and wellbeing and reduce the burden of NHS resources required to care for them post operatively. This research aimed to explore practitioners’ experiences of and strategies for providing intrapartum care to obese women.

**Method:**

A qualitative methodology was adopted, focus groups and individual interviews were conducted with health professionals. Audio recordings were transcribed verbatim and data analysed using a framework approach.

**Results:**

Twenty-four health professionals participated; Six Consultant Obstetricians two Consultant Anaesthetists and 16 midwives. Three key themes emerged from the data: medicalisation of obese birth; promotion of normal obese birth; and the complexities and contradictions in staff attitudes and behaviours. The overall interpretation is that positive approaches to obese birth offer opportunities to promote normal birth. However, many health professionals find the provision of intrapartum care to obese women challenging, and attitudes and behaviours towards the promotion of normal birth are heterogeneous, complex and contradictory.

**Conclusion:**

The care of obese women during labour is generally medicalised and focussed on the associated risks. However, although there are conflicting views on how to care for obese women, some practitioners do strive to promote normality and optimise the potential for normal birth by challenging current practices and utilise some ‘interventions’ in order to facilitate normality and mobility during childbirth. Obesity is a major and growing health problem and a major cause of morbidity and mortality for pregnant women. It is essential that more positive proactive guidelines are available to maximise normal birth if the postnatal health of obese women is to be improved.

**Electronic supplementary material:**

The online version of this article (doi:10.1186/s12884-015-0673-2) contains supplementary material, which is available to authorized users.

## Background

Obesity is emerging as one of the greatest health problems in the developed world. Rates of obesity vary, with the highest rates currently in the Pacific Islands (45–75 %) and Kuwait (42 %). In the United Kingdom (UK) approximately 27 % of adults are currently obese [1].

Obesity, defined as a body mass index (BMI) over 30, affects one-fifth of women in the UK [2] with the prevalence increasing in both the general and pregnant population. Obesity is a significant contributor to maternal deaths and women with a high BMI remain over-represented in all maternal deaths [3, 4]. Obese pregnant women also have a higher risk of a number of pregnancy complications, including miscarriage, pre-eclampsia, gestational diabetes, fetal macrosomia and stillbirth [5–14]. Maternal obesity can have a direct influence on mode of birth and postnatal morbidity. Obese women are more likely to receive medical interventions, including caesarean delivery and general anaesthesia [5]. The rate of induction of labour is reported to be doubled for obese pregnant women, compared to non-obese women [15, 16]. Delay in the first stage of labour is significantly more common [17–20], with the risk ranging from 1.5 times to 3 times more likely. Obese women also have a significantly increased risk of caesarean section of between 2-fold to more than 3-fold [13, 15, 18, 19, 21–25], with the most common reason for caesarean section being delay during the first stage of labour, even after augmentation with oxytocin [17–19]. Caesarean section also carries additional risks for obese women and has a considerable impact on postnatal morbidity, with maternal obesity being an independent risk factor for post-caesarean infections [26].

Little is known about the benefits of delaying a decision for caesarean section to promote normal birth from women’s or clinicians’ perspectives. In 2011–12, there were 813,200 births in the UK [27]. In the UK the National Institute for Health and Care Excellence (NICE) have published guidance on caring for low-risk women and their babies during the intrapartum period. This offers evidence-based advice on the care of healthy women with uncomplicated pregnancies at low-risk of developing complications during labour and birth [28].

Additional publications specifically focussed on maternal obesity provide advice on the clinical management of obesity during pregnancy [29, 30]. They emphasise medical care for obese pregnant women, with the primary aim to promote safety. Whilst acknowledging that safety is of paramount importance, increasing medical intervention for these women may also increase the risk of complications, which could itself have detrimental effects. For example, the use of continuous electronic fetal monitoring has shown an association with an increased rate of both cesarean delivery and operative vaginal delivery [31] and caesarean section subsequently carries an increased risk of postpartum haemorrhage [7] and post-operative infection [26].

In the UK maternity care is provided through a network of birth settings, either consultant-led or midwifery-led. Midwives are involved in the provision of care to pregnant women during pregnancy, during labour and birth and in the postnatal period, in all birth settings. A telephone survey of 41 hospitals, conducted by the lead author prior to the commencement of this study explored to what extent guidelines for the intrapartum care of obese women were available in maternity hospitals across the UK. That survey found that the majority of hospitals had clinical guidelines for the obstetric management of obese women during the intrapartum period, however, only a small number made reference to midwifery care during labour. The majority of the content of the guidelines focussed on obstetric care, for example, recommending that birth take place on the consultant-led unit, the anaesthetist be informed on arrival to the labour ward, and continuous electronic fetal monitoring during labour. Only three guidelines made any direct reference to normal birth. The dual problem of increasing birth rates and increasing rates of obesity makes this a significant problem for women’s health and for NHS resources. It is imperative to improve both obese women’s experience and outcomes of childbirth. However, there is evidence that midwives experience difficulties supporting obese women to have a more normal, physiological birth. Midwives find caring for obese women during labour challenging, in particular, the loss of ‘normality’ and the physical difficulties of providing care [32]. Obese pregnant women themselves report negative experiences of maternity care overall, experience feelings of guilt and many report prejudice and negative attitudes from staff when accessing maternity care [33, 34].

This study aimed to explore practitioners’ experiences of providing intrapartum care to obese pregnant women. The specific objectives were;

- To obtain practitioners’ experiences of caring for obese pregnant women,
- To identify the issues that practitioners face when caring for obese pregnant women,
- To identify how these issues impact on patient care,
- To identify possible solutions that could decrease the impact on care.

## Method

The study used a qualitative methodology. Focus groups and individual interviews were conducted with health professionals who provided antenatal and/or intrapartum care to obese women, including Midwives, Obstetricians and Anaesthetists. The study was carried out in two National Health Service Hospitals, one in England, a large tertiary unit, with an annual birth rate of approximately 8000 and one in Scotland, a district general hospital with an annual birth rate of 5000 births. These two hospitals were chosen because they both served a large obese population and were willing to participate in the research. The local guidance for the care of obese women was similar at both hospitals. Ethical approval was gained prior to commencement of the study from the Health Research Authority, National Research Ethics Service Committee (12/NW/0631).

All midwives who provided antenatal and/or intrapartum care to obese women were sent an information pack about the research and were asked to indicate whether or not they were willing to participate using a reply slip. The response rate 30 %. Consultant Obstetricians and Anaesthetists were also sent a research information pack. This was followed up with a telephone call to ascertain if they wished to participate and arrange a mutually convenient time for an interview.

Focus groups were conducted with midwives and individual interviews were carried out with Obstetricians and Anaesthetists. Focus groups were chosen as an appropriate method for midwives as they usually work in teams and focus groups allow for more discussion and a larger sample size. Obstetricians were interviewed individually as they generally work individually and interviews were more convenient to arrange around obstetricians workload. Midwives who were unable to attend the focus groups, but wished to participate, were interviewed individually. An interview guide was used to guide the discussions and they were audio-recorded with consent (see Additional file 1).

**Fig. 1:**
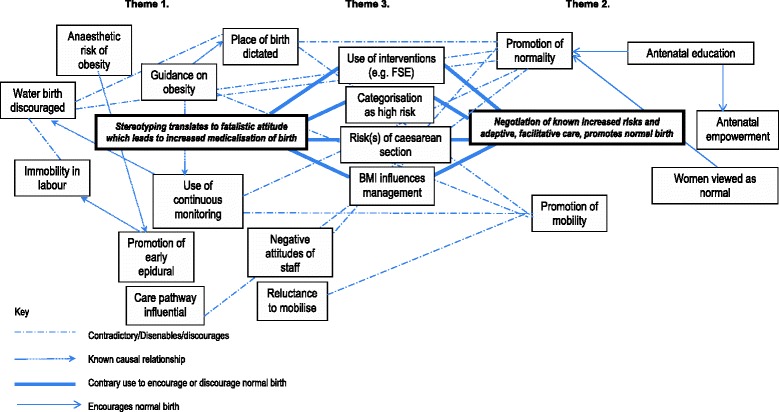
Conceptual Framework

The audio-recordings were transcribed verbatim by a professional transcription service. Transcripts were checked for accuracy against the audio recordings by AK. Data was analysed using a framework approach [35]. This involved a five stage process of (1) familiarisation, where the transcripts and study notes are read several times to identify recurrent themes; (2) identification of a thematic framework, where the main themes and sub-themes are sorted into a detailed framework; (3) indexing, where the thematic framework is applied to the data in order to label or index it; (4) charting, where the data is grouped according to the part of the framework they relate to, creating a series of charts and (5) mapping and interpretation, where the charts are used to define concepts, create typologies and identify associations between the themes. Two authors (AK and HC) undertook the lengthy processes of familiarisation and identification of an initial thematic index. This thematic framework was subsequently refined by two authors (AK and CK) during the processes of charting, mapping and interpretation. Consensus was reached through discussion. Figure 1 depicts the relationship between emergent themes (and sub-themes) that lead to the overarching conceptual framework, which comprises three themes resulting in two key propositions. Table 2 shows the thematic framework of the findings

## Results

Twenty-four health professionals participated across the two hospitals. Six Consultant Obstetricians and two Consultant Anaesthetists were interviewed individually. A total of 16 midwives participated in either a focus group or an individual interview, all of whom were regularly provided intrapartum care to obese women. See Table 1 below.

**Table 1:** Participant profile

	England	Scotland
Obstetrician	3	3
Anaesthetist	1	1
Midwife	10	6
Focus Group 1	3 midwives	-
Focus Group 2	4 midwives	-
Focus Group 3	-	4 midwives
Focus Group 4	-	2 midwives
Interviews	3 midwives	-

Table 2 shows the thematic framework of the findings. The overall interpretation ‘Different approaches to obese birth offer opportunities to promote normal birth’ was underpinned by three key emergent themes; Medicalisation of obese birth; promotion of normal obese birth; and the complexities and contradictions in staff attitudes and behaviours. These three themes and their sub-themes are presented in Table 2, with examples of some of the codes used during the analysis and some excerpts from the data.

**Table 2:** Thematic Framework

Interpretation: Different approaches to obese birth offer opportunities to promote normal birth					
Theme 1		Theme 2		Theme 3	
Medicalisation of obese birth		The promotion of normal ‘obese’ birth		Complexities and contradictions in staff attitudes and behaviours	
Place of birth	Place of birth impacts on mobility	Antenatal education	Importance of information-giving antenatally	Use of fetal scalp electrodes	FSE used to aid mobility
*“We had a woman who wanted to sit on a ball because she was a home delivery, but had to be continuously monitored and they (staff) were unhappy to do it at first”*		*“I think we should be educating them about mobility and being mobile and trying to get them to the MLU”*		“*I would preferably, be able to monitor the babe, put the FSE on, to make sure that if she wanted, she could be mobile to help the labour as well”.*	FSE viewed as an intervention by some but used to promote mobility by others
	Normailty influenced by place of birth		Antenatal education about mobility		
Negative attitudes of staff	Negative attitudes about women’s size	Promotion of normality during labour	Acknowledge risk but promote normality same as anyone else	Risk of caesarean section	Risk of caesarean can influence care
“*And the delivery of those patients, I think it’s probably looked at negatively by the midwifery staff as well to an extent, because they are overweight they see them as ‘oh, this person’s going to be a problem’*		*“We should be treating them the same, if not more so promoting normality”*		*“I think people tend to play safe. I don’t think I personally would agree with that….It’s best to have a normal delivery and if it can be, you know, pushed to that stage, without taking much risk, I will do that. Rather than doing something, like ding a section for example”*	Not all obese women have a caesarean
	Caring for obese women viewed negatively		Pro-active approach to normality		
Challenges monitoring fetal heart	Technically difficult monitoring fetal heart	Promotion of mobility during labour	Promote mobility regardless of size	BMI influencing clinical management	BMI may influence decision-making for caesarean section
*“I just had to stand there and I was trying to get something and half the time you didn’t know if it was maternal pulse, it was very difficult”*		*“I think basic care should be managed exactly the same. Like, cos any woman should be mobile in labour, you know, regardless of what they weigh”*		*“I don’t feel that I do, but I do feel that some people probably make decisions where the lady’s weight influences their decisions”*	
					BMI may influence decision making positively
	Fetal heart monitoring is difficult				
Reluctance to mobilise	Obese women less mobile in labour			Classification as high risk	High risk classification can be detrimental
*“I think they’re generally more difficult. They’re more reluctant”*				*“I think putting somebody in a high risk category actually doesn’t do anybody any favours because then people tread very carefully and they start to think ‘oh God, she’s high risk……I better make sure that nothing wrong happens here’”*	
	General reluctance to mobilise				Women view themselves as ‘normal’
Discouragement of use of water	Water birth contraindicated because of size				
*“Because at the moment women are excluded from water birth aren’t they, who have a BMI over 35”*					
	Water birth not an option				

The relationships between emergent themes (and sub-themes) are shown in Fig. 1. The lines depicting causation were informed by what is known in existing literature, with the final iteration originating directly from the data. These led to the overarching conceptual framework comprising of two key propositions. First, the routine stereotyping of women categorised as obese leads to fatalistic staff attitudes and a pre-emptive medicalisation of birth as abnormal. Secondly, the care of women categorised as obese can be facilitative and adaptive to promote normal birth whilst negotiating known increased risks. These two propositions co-exist and are held in tension, but at the same time are not mutually exclusive, or associated with a particular professional group, leading to an element of fluidity. Moreover, as evident in theme 3 (middle ground) both could afford opportunities to promote normal birth.

## Medicalisation of ‘*obese’* birth

### Place of birth

The current guidance [29] states that women with BMI over 35 should give birth in a consultant led unit and this was echoed by the midwives.

*“Nationally the recommendation is that anyone with a BMI of 35 or more should be in consultant-led” (M/W FG)*

Whilst acknowledging the guidance, some midwives felt that although women with a raised BMI were ‘not allowed’ to give birth on the Midwifery Led Unit (MLU), they did sometimes achieve a normal birth.

*“I had a woman that had a raised BMI that wasn’t allowed on the MLU because of a certain cut off that they had a long time ago, who came in, mobilised and pretty much delivered herself” (MW Int)*

### Negative attitudes of staff

The attitudes of staff towards obese women was discussed by both obstetricians and midwives and it was acknowledged that caring for obese women, particularly during the intrapartum period was viewed negatively, with many staff displaying a negative attitude towards the prospect of providing care.

*“The minute you see somebody come through delivery suite who’s very large you hear people ‘oh, I don’t want to look after her, don’t give her to me…..so immediately they are negative…..so I don’t know how they’re going to be when they get the woman in the room” (MW Int)*

One midwife expressed concern as to how the negative attitudes of staff affected the women they were caring for.

*“They’re already feeling negative about caring for her, so I don’t know how that would then come across to the woman….” (MW Int)*

Several reasons were suggested for this common attitude, and included the physical difficulties that are encountered for example:

### Challenges monitoring the fetal heart

The practice of using continuous electronic fetal monitoring when caring for obese women during labour is common and was discussed and challenged by both midwives and obstetricians. Many practitioners were not able to recall any evidence for this use of continuous fetal monitoring.

*“I can’t remember it [obesity] being one of the things that we put down as an indicator for continuous monitoring” (Obs Int)*

*“Continuous monitoring…I don’t think there is any evidence that says so” (Obs Int)*

The use of continuous monitoring during labour was viewed as very restrictive for women and it was felt that this was detrimental to the promotion of normality and mobility during labour. Midwives felt that continuous fetal monitoring was more likely to restrain a woman to a bed during labour and medicalise their labour.

*“…Continuous monitoring, that’s going to put somebody on a bed before they’ve even started” (MW FG)*

The challenges of both intermittent auscultation and continuous monitoring were acknowledged, with the need for the use of ultrasound to locate a fetal heart being common.

*“Even intermittent auscultation is more difficult for the midwives to physically perform when the women are obese….You end up having to do ultrasounds to locate the heart….” (Obs Int)*

One midwife described the difficulties she had performing continuous monitoring, being unable to confidently distinguish between the fetal heart rate and the maternal pulse rate.

*“I just had to stand there and I was trying to get something and half the time you didn’t know if it was maternal pulse….it was very difficult” (MW FG)*

### Women’s reluctance to mobilise

One of the major difficulties encountered by midwives when caring for obese women during labour was motivating them to be mobile during labour and have an active birth, with many women wishing to be relatively immobile during their labour. They found motivating them to get off the bed and move around to be particularly challenging.

*“It’s hard to get them up, it’s hard to move them about” (MW FG)*

The physical size of the women and the extra effort that it took to be able to mobilise was seen as a reason for the reluctance.

*“I think sometimes that the very biggest ladies do tend to be a little bit more reluctant to do that [mobilise], only because you can see it just takes so much more effort for them to move” (MW Int)*

However, some midwives recognised that although obese women were more likely to be less mobile during labour, they also acknowledged that some obese women were embarrassed that they found it more difficult to mobilise and even though they were less mobile, it was not necessarily through choice.

*“I don’t think they like being immobile. I think they find it embarrassing” (MW FG)*

### Discouragement of water birth

Finally, the discouragement of hydrotherapy and water birth for obese women was an important factor that contributed to the medicalisation of obese birth.

*“No I don’t think they are allowed in the pool” (MW FG)*

The reasons for obese women being discouraged from using hydrotherapy for either analgesia or birth were commonly related to manual handling risks, in particular the need to evacuate the pool in an emergency

*“I had a large lady a few weeks ago and she said to me ‘oh I was told I could have a pool birth’ and I said ‘no, because it would be difficult to hear your baby and to get you out in an emergency” (MW FG)*

Contrary to this, the multiple benefits of hydrotherapy for obese women were acknowledged, in particular the benefits of relative weightlessness and buoyancy to aid mobility during labour.

*“One of the difficulties that people with high BMIs have is difficulty in changing positions….and to have somebody like that buoyant in water takes all the pressure off their pelvis……” (Obs Int)*

*“That’s the difficulty with water birth isn’t it? Because they are the ideal sort of group to benefit….the weightlessness” (MW FG)*

## The promotion of normal *‘obese’* birth

Contrary to the fatalistic attitudes of some midwives and obstetricians towards obese women in labour, the promotion of normal birth was widely discussed.

### Antenatal education

Antenatal education was viewed as a key factor in the promotion of normal birth. Informing women during pregnancy about normal birth and preparing them for labour was viewed as a fundamental part of antenatal education, in order to make women aware of what to expect.

*“It’s also about education isn’t it? So that she knows what’s coming, that she needs to be doing all the right things” (MW FG)*

Some midwives spoke of the importance of educating women about mobility during labour, in order to prevent immobility on beds during labour.

*“I think we should be educating them about mobility and about being mobile and trying to get them to the MLU” (MW Int)*

### Promotion of normality

Promoting normality during labour in an integral part of the midwife’s role, regardless of the obstetric, medical or demographic history of the woman. The encouragement and promotion of normal birth was viewed as fundamental in the care of obese women. One midwife, whilst acknowledging the guidance, felt it was the midwife’s role to actively promote normality birth for obese women, in order for them to optimise their chance of normal birth.

*“I think we should be encouraging them to have more of a normal birth” (Obs Int)*

*“Rather than sitting back and just saying the guidelines say this; let’s encourage it, let’s promote it” (MW FG)*

### Promotion of mobility

Similarly, the promotion of mobility during labour was acknowledged as an essential part of intrapartum care for obese women.

*“I’d try to keep her either active on a ball or active over the side of the bed…I would keep her as upright as possible” (MW Int)*

Midwives felt that mobility has benefits for all women in labour, with obese women in particular, benefitting significantly from being mobile during labour and birth in order to overcome the risks of prolonged labour and operative birth.

*“I think possibly if you keep obese pregnant women upright and mobile you’re probably going to get a better outcome, you’re probably going to get a nice delivery” (MW FG)*

*“I think it wouldn’t be difficult to promote, I think it’s the best thing to promote mobility in that population, they need to be upright” (MW FG)*

## Complexities and contradictions in health professionals’ attitudes and behaviours

The final theme is that of the complexity surrounding the conflicting attitudes to some of the associated risks of obesity and the use of some medical technologies when caring for obese women during labour and birth. Several contradictions existed towards the use of medical interventions and the associated risk of caesarean section for obese women, as these were viewed as either prohibitive to or facilitative of normal birth.

### The use of a fetal scalp electrode

The use of a fetal scalp electrode (FSE) to monitor fetal heart rates in obese women was widely discussed and there were two very distinct attitudes towards their use in practice. The use of an FSE was commonly seen as a medical intervention associated with high-risk care and could potentially prohibit the promotion of normality

*“Unless they’ve put an FSE on, which is very interventional really, isn’t it, when you’re trying to promote normality” (MW FG)*

There was also a common assumption that the application of an FSE would lead to a higher incidence of immobility during labour and it was often cited as a reason why women were not mobile in labour.

*“They tend to end up with fetal scalp electrodes on and you’re automatically medicalising labour in a group of women that we know, probably don’t labour as well, so would benefit greatly from being more mobile” (Obs Int)*

*“Although theoretically if you’ve got a scalp clip on you are supposed to be more mobile, but I don’t necessarily see that transferring into practice” (Obs Int)*

Contrary to the negative attitudes surrounding the use of FSE, some midwives and obstetricians viewed their usage positively and whilst acknowledging it as an intervention, felt that they could be used as a catalyst for normal birth, in particular, saw the use of an FSE as an effective way to increase mobility.

*“We tend to use FSEs quite a lot if we’ve got somebody that’s on continuous monitoring, so that we can get them up” (MW FG)*

*“Put an FSE on, to make sure that if she wanted, she could be mobile” (MW Int)*

This was because it is a more accurate way of recording the FH compared to an abdominal transducer and did not lose the contact when women were mobile.

### Risk of caesarean section

The risks of and the decision for caesarean section were discussed widely amongst obstetricians. Some obstetricians reported a much lower threshold for making a decision for caesarean section than they would with a non-obese woman, basing decisions on the safety of the woman. One obstetrician felt that decisions to proceed to caesarean section during labour varied widely between each individual obstetrician, with some obstetricians trying to avoid the need to perform a caesarean section, because of the increased risks associated with operative birth.

Conversely it was felt that some obstetricians make decisions for caesarean section based on the time of day and the availability of consultant staff, with decisions made earlier than they would normally do for a non-obese woman.

*“I do feel that some people probably make decisions where the lady’s weight influences their decision. So whether they don’t do a caesarean as soon as they should do because they are trying to avoid doing a caesarean ….or they do it sooner than they should do because they want to do it when the consultant staff are available” (Obs Int)*

Interestingly, one obstetrician suggested that they would in fact allow more time for an obese woman to labour before making a decision for caesarean section, in order to avoid the need for caesarean section and the associated risks, with an aim to facilitate normal birth.

*“No I think we’d give it the same, in fact I might even give it longer, it’s not much fun doing a caesarean section on a very obese patient, so no, I don’t think we jump in early” (Obs Int)*

### BMI influencing clinical management

The influence that a woman’s BMI had on the clinical management of labour and birth was discussed by a number of obstetricians. This was another area that demonstrated the presence of contrasting views, with maternal BMI seen to influence clinical management both in the prohibition and facilitation of normal birth.

*“I do feel that a woman’s size can influence your management and it’s very difficult to do that because obviously the woman’s safety is paramount, but it probably does then affect the way you manage her” (Obs Int)*

The attitude towards obese woman directly influenced the decision making process, with perceptions that obese women could be potentially problematic and therefore had significantly influenced clinical decision-making

*“I would suspect it is a way in which we manage their care and I suspect we do see them as a problem…” (Obs int)*

### Classification as ‘high-risk’

Obese, pregnant women are currently widely regarded as ‘high risk’ in obstetric terms, because of the higher likelihood of a number of antenatal, intrapartum and postnatal complications, but this can significantly impact on the management of intraprtum care. It was felt amongst Obstetricians that classification as ‘high-risk’ is appropriate for women with raised BMI because of the increased risk of intrapartum complications.

*“They are at higher risk of complications of labour, so I would think yes, yes they are [high risk] (Obs Int)*

Interestingly, although some obstetricians and midwives did not disagree that obese pregnant women were at higher risk of complications, some felt that labelling them as ‘high risk’ was particularly negative and could be detrimental to their care and ultimately their chances of normal birth

*“I think putting somebody in a high risk category actually doesn’t do anybody any favours” (MW FG)*

Some midwives felt that this classification directly affected the woman’s attitude and motivation for normal birth.

*“I think a lot of them come in and they’ve been told, the risk is this, the risk is that, so they have the mindset, then that’s what’s going to happen to me” (M/W Int)*

Whilst others acknowledged that although the risks were higher for obese women, women should be encouraged to have a positive attitude to birth and ultimately empowered to try and overcome the risks and achieve a normal birth. The way the information on the associated risks was delivered was seen as a crucial factor in this.

*“I know the risks are much higher, but they don’t all and if you get it across to people that, think positively, you know” (MW Int)*

## Discussion

The aim of this study was to explore practitioners’ experiences of providing intrapartum care to obese pregnant women. Our findings described the experiences of health professionals, when caring for obese women during labour, including the medicalisation of obese birth, the promotion of normality for obese women and the complexities of health professionals’ behaviour surrounding obese women in labour.

### Promotion of normal birth

In the UK, successive policy documents have explicitly promoted normal birth for healthy women and their babies for over two decades [36, 37]. Our earlier survey found the promotion of normal birth is not included in the majority of clinical guidelines for the care of obese pregnant women. However, despite this, midwives and obstetricians who participated in this study described the promotion of normality and normal birth as an integral part of their role when caring for obese women during labour. Antenatal education for obese women was viewed by midwives as an essential aspect in this, in order to allow women to have realistic expectations of labour and birth and promote normal birth. This is supported by Schott & Priest who suggest that if you prepare women for the physical and emotional realities of labour and birth, they will be confident that what they are actually experiencing is normal and are more equipped and able to cope [38]. The national guidance on obesity recommends that women should be informed of the risks associated with obesity during pregnancy and advised on how to minimise them. It states that women should be made aware of the potential difficulties with caesarean section, but offers no guidance on how to minimise the need for caesarean section [29]. This is not just specific to obese women, as currently there is no guidance available on minimising the risk of caesarean section, regardless of Body Mass Index, however, all pregnant women are offered the opportunity to attend antenatal education in order to prepare for labour and birth.

The promotion of mobility during labour was viewed as an essential aspect of their care, in order to minimise the associated risks of prolonged labour and operative birth and midwives felt that if women were advised during the antenatal period of the importance of mobility during labour, they would be more likely to mobilise from the outset. Mobilisation during labour is widely acknowledged as a way of optimising the likelihood of normal birth [39, 40] and this is reflected in the practices and attitudes described by the midwives, who viewed it as an integral part of their care, despite the challenges faced with this population. Interestingly, Singleton & Furber found that although midwives advocated the need for mobilisation, they felt obese women were not able to remain mobile during labour because of the associated risks of obesity during labour, which restricted their options [32].

In order to support and encourage mobilisation during labour and the promotion of normal birth, techniques used to promote normal birth were described. Techniques such as the use of an FSE to allow women to be fully mobile during labour, whilst continuously monitoring the fetal heart rate are commonly utilised with obese women, with many seeing their usage as a positive intervention and a potential catalyst for normal birth. However there was conflicting views of this practice, with some practitioners viewing the use of an FSE as a medical intervention, with the potential to inhibit mobility and normality. The wide spread use of FSE in obese women reflects the national guidance that suggests that fetal scalp electrodes should be utilised if adequate fetal heart monitoring proves challenging [29]. Many midwives adopted this guidance into their practice and whilst acknowledging the use of an FSE to be an intervention, they utilised this method of fetal monitoring to prevent women becoming immobile in order to adequately monitor the fetal heart.

### Conflicting attitudes

The apparent lack of consensus surrounding the clinical management of labour and birth for obese women, particularly caesarean section, is interesting. Some obstetricians reported a much lower threshold for making a decision to proceed to caesarean section than they would with a non-obese woman, whilst other obstetricians reported actively trying to avoid a caesarean section because of the increased associated risks of operative birth in this population.

It could be argued that the increased risk of caesarean section in obese women [18–20], should be a used to encourage the facilitation of normal labour and birth. The most common reason for caesarean section is delay during the first stage of labour, even after augmentation with oxytocin [17–19] and therefore, the facilitation of mobility during labour and the use of mobility aids may prevent delay during labour and therefore the need for caesarean section. Some obstetricians reported trying to avoid a performing a caesarean section on an obese woman, unless absolutely necessary and would often allow more time for labour to progress before making a decision that operative delivery was necessary. The facilitation of mobility during labour, would minimise the risk of delay and therefore the need for caesarean section [41].

At the same time it was evident that negative attitudes towards obese women were directly influencing clinical decision making processes with obese women commonly viewed as problematic and decisions to proceed to caesarean section were made a lot earlier compared to non-obese women, in order to attempt to minimise additional intrapartum or postnatal complications. In this situation, it could be argued that the increased risk of caesarean section encouraged obstetricians to proceed to caesarean section sooner than they would with a non-obese woman, preventing women from optimising their chance of normal birth. Interestingly the negative attitudes towards caring for obese women was attributed to colleagues. None of the participants admitted to displaying negative attitudes themselves.

### Medicalisation of birth

The medicalisation concept has been variously theorised in medical sociology in general [42, 43] and in relation to childbirth in particular [44, 45]. Whilst early medicalisation of childbirth literature was almost exclusively critical, by the mid-1980’s there was increasing recognition of how these processes are co-constituted by clinicians’ and women themselves. Over the last two decades there has been a dearth of medicalisation theorising in relation to childbirth [46]. The present study highlights the need to revisit the medicalisation concept in relation to different groups of women’s contemporary experiences of childbirth. This study challenges the old medicalisation of childbirth dichotomy between medical and natural (midwifery) models of childbirth for all women. Our findings demonstrate the complex and contradictory use of technology to promote normal birth by midwives and obstetricians, specifically for obese women.

The medicalisation of obese women during labour and the challenges to providing care was discussed. Some participants expressed the view that obese women should be viewed as ‘high-risk’ and the care should be medicalised, reflecting the UK national guidance. However, some midwives expressed an opposing view and viewed the promotion of normality to be an integral part of the care they provide to obese women, challenging the medicalisation of care advised in the national guidance. It was widely acknowledged that continuous monitoring of the fetal heart was one of the biggest challenges and led to the medicalisation of labour and birth. Many practitioners challenged this practice and were unable to confidently recall the evidence on which this practice is based. The national guidance on the management of obesity during pregnancy (page 12) is quite ambiguous, suggesting that fetal heart rate monitoring in obese women can be challenging and ‘close surveillance is required with recourse to fetal scalp electrode or ultrasound assessment of the fetal heart if necessary.’ [29], however, it does not explicitly state that continuous monitoring is necessary. The accepted practice of continuous monitoring could be questioned and challenged as it has a significant impact on the management of labour and may lead to unnecessary intervention and medicalistion of birth.

The discouragement of water birth for obese women was viewed as a contributing factor to the medicalisation of care for obese women. The reasons for obese women being discouraged from using hydrotherapy were stated to be related to manual handling risks, but the multiple benefits were also acknowledged, including the increased ability to stay mobile during labour. Swann & Davies suggest that the advantages of using water in labour are equally, if not more applicable to obese women and include the use of water as a mobility and positon aid, increasing the pelvic outlet and reducing the potential for delay during labour [47]. Difficulties monitoring the fetal heart rate are commonly cited as reasons for discouraging water birth in obese women, Swann & Davies suggest that the use of waterproof telemetry could overcome this difficulty and with the increasing availability of wireless telemetry, this could also be utilised to facilitate the use of hydrotherapy for women who require continuous electronic fetal heart monitoring [47]. However, as discussed earlier, the common practice of continuous fetal heart monitoring for obese could be challenged, as it could be argued that the evidence on which this practice is currently based is ambiguous.

The need to promote normal birth for obese women, including antenatal education, the promotion of mobility and the need to minimise the risk of caesarean section and the challenges to providing care to obese women, including the practice of continuous monitoring and the discouragement of water birth was widely discussed and reported. However, Singleton & Furber suggest that instead of practitioners striving to encourage normal birth, it may be more appropriate to advocate ‘optimal care’, as this aims to achieve the best possible birth for the women, whilst acknowledging the associated risks [32].

## Strengths and limitations

This was a relatively small survey, including 24 health professionals. Whilst the findings are not intended to be generalizable, they resonate with anecdotal experiences in practice as well as being supported by the limited literature that exists in this area [32]. However, the sample was obtained from two hospitals, allowing a varied sample to be obtained, including England and Scotland. A further strength is that it included midwives, obstetricians and anaesthetists and so allowed experiences from all health professional groups to be obtained. Additionally, it involved systematic data collection and analysis using the Framework approach by trained qualitative researchers. The analysis was conducted with rigour, with two authors (AK, CK) identifying and corroborating emerging themes and all authors reaching a consensus on the final interpretation.

## Conclusion

This work has clearly demonstrated that the care of obese women during labour is often medicalised and focussed on the associated risks. However, although obese women are sometimes stereotyped and there are conflicting views on how to care for obese women, some practitioners do strive to promote normality and optimise the potential for normal birth by challenging current practices and utilise some ‘interventions’ in order to facilitate normality and mobility during childbirth. Obesity is a major and growing health problem and a major cause of morbidity and mortality for pregnant women. It is essential that there are more proactive guidelines to maximise opportunities for normal birth if the postnatal health of obese women is to be improved.

## Additional file

Additional file 1:
**Interview Guide.** (PDF 5 kb)

## Abbreviations

BMI: Body mass index
CMACE: Centre for Maternal and Child Enquiries
CU: Consultant unit
DGH: District General Hospital
DH: Department of Health
MLU: Midwifery Led Unit
RCOG: Royal College of Obstetricians and Gynaecologists
UK: United Kingdom
