# Supplementary material for: Obesity and normal birth: A qualitative study of clinician’s management of obese pregnant women during labour
Source: BMC Pregnancy Childbirth. 2015 Oct 12;15:256. doi: 10.1186/s12884-015-0673-2 (PMC4603577; doi:10.1186/s12884-015-0673-2)
Supplement: Additional file 1: — Interview Guide. (PDF 5 kb) [file 12884_2015_673_MOESM1_ESM.pdf]

Interview Guide (Version 1. 1/11/10)

1) Have you experienced any particular issues (or problems) when caring for an obese woman during labour? What were these?

2) How did these affect the care you provided/were able to provide?

3) Can you think of any possible solutions to these issues? What has worked well in caring for obese women?

4) Do you think intrapartum care of obese women should be managed differently to non-obese women?

5) Do you feel that you have enough information or training in the care of obese women? Is there any information/training you would like to receive in this area?
